# Efficacy of Infraclavicular Brachial Plexus Block Alone Versus Combination With Suprascapular Nerve Block in Patients Undergoing Shoulder Surgeries: A Single-Blind, Randomized Trial

**DOI:** 10.7759/cureus.52961

**Published:** 2024-01-25

**Authors:** Parul Saxena, Manish K Singh, Manoj K Chaurasia, Sarita Singh

**Affiliations:** 1 Department of Anesthesiology, King George's Medical University, Lucknow, IND

**Keywords:** post-operative pain, shoulder surgeries, regional anaesthesia, suprascapular nerve block, infraclavicular brachial plexus block

## Abstract

Background and aim

The regional anesthesia technique is commonly used for upper extremity surgery as an alternative to general anesthesia. The study aimed to compare the efficacy of infraclavicular brachial plexus block (BPB) and a combination of infraclavicular brachial plexus block with suprascapular nerve block for postoperative analgesia in patients undergoing shoulder surgeries.

Method

A total of 62 patients of both sexes with the American Society of Anaesthesiologists (ASA) physical status I/II/III, aged between 18 and 65 years, and undergoing shoulder surgery, were included in this prospective, single-blinded, randomized controlled trial. Patients were equally allocated into two groups: 31 in group A and 31 in group B. After pre-anesthetic evaluation, the purpose and protocol of the study were explained to patients, and informed consent was obtained. Thirty-one patients in group A were given infraclavicular brachial plexus block using 30 ml 0.375% bupivacaine while 31 patients in group B were given a combination of infraclavicular brachial plexus block using 30 ml 0.375% bupivacaine and suprascapular nerve block using 5 ml 0.375% bupivacaine. Blocks were given using ultrasound guidance and a peripheral nerve stimulator; the suprascapular block was given in the sitting position while the infraclavicular block was provided in the supine position. General anesthesia was administered in the operation theatre in the supine position after the administration of blocks. The pain was assessed using the visual analog scale (VAS) and the satisfaction score was assessed by the numeric rating scale (NRS). The Mann-Whitney U test was applied for comparison of pain between the two groups. The chi-square test was utilized for comparing the categorical variables.

Result

The postoperative pain was significantly lower (p<0.001) in group B as compared to group A at all the periods of observation, i.e., 0h (2.77±0.72 vs. 5.42±0.77), 6h (3.89±0.70 vs. 5.94±0.73), 12h (5.66±0.93 vs. 6.58±0.88), and 24h (6.16±0.80 vs. 6.74±0.90). These findings illustrate that group B patients who received a combination of infraclavicular brachial plexus block and suprascapular nerve block for shoulder surgeries had better pain relief than group A patients who received only the infraclavicular approach. The mean NRS score of patient satisfaction in group B (7.26±0.58) was significantly higher (p<0.001) in comparison to group A (6.16±0.64). Diaphragmatic palsy was observed in only one case in group A and none in group B. No other complication was observed in any of the patients during the study period.

Conclusion

The combination of infraclavicular brachial plexus block and suprascapular nerve block displays a positive postoperative analgesic profile with less usage of rescue analgesic doses and better patient satisfaction after shoulder surgery.

## Introduction

Surgical procedures for shoulder instability, like rotator cuff repair, shoulder arthroscopy, and subacromial decompression, are routinely done. Anesthesia and analgesia for these surgical procedures are provided by general anesthesia, regional anesthesia, or a combination of general and regional anesthesia. Shoulder surgery is frequently associated with high levels of postoperative pain, which may require analgesia with opioids for several days [1]. Postoperative pain management after shoulder surgery is a big challenge due to the side effects of opioids.

Brachial plexus block (BPB) around the clavicle has been commonly used for upper extremity surgery as an alternative to general anesthesia. The interscalene brachial plexus block is widely used and considered the most effective method for managing intraoperative and postoperative pain in patients who are undergoing shoulder surgery. However, in recent years, there has been increasing research into alternatives to the classic interscalene block due to its wide spectrum of complications.

The innervation of the shoulder joint is provided by several nerves [2]: the subscapular, axillary, lateral pectoral, and suprascapular nerves. A single injection at the cord level during the infraclavicular block can effectively block the subscapular, axillary, and lateral pectoral nerves while the suprascapular nerve needs to be separately blocked. The infraclavicular block offers several advantages, as it provides thorough anesthesia of the upper limb by targeting the brachial plexus, where the three cords closely accompany the axillary artery. Injuries to vital neurovascular structures in the neck and the incidence of inadvertent pleural puncture are lower than with the interscalene and supraclavicular approaches. Infraclavicular block can be performed with the patient comfortably positioned, as it does not require abduction of the arm at the shoulder [3]. The suprascapular nerve provides sensory innervation to the acromioclavicular and glenohumeral joints and motor innervation to the supraspinatus and infraspinatus muscles. By targeting this nerve, it is possible to achieve shoulder pain relief without affecting the phrenic nerve [4]. This procedure is usually performed as a diaphragm-sparing alternative to the interscalene brachial plexus block.

Theoretically, the infraclavicular brachial plexus block targets the posterior and lateral cords, thereby anesthetizing the axillary nerve (which supplies the anterior and posterior shoulder joint) as well as the subscapular and lateral pectoral nerves (both of which supply the anterior shoulder joint) while the suprascapular nerve block (SSNB) anesthetizes the posterior shoulder. Martinez et al. successfully combined an infraclavicular brachial plexus block with a selective suprascapular nerve block for a humeral head repair in a study of a patient with respiratory failure [5]. Anatomically, it seems plausible that an infraclavicular brachial plexus block may block the axillary, subscapular, and lateral pectoral nerves [6]. Surprisingly, this case report did not lead to further examination of this approach until recent papers suggested it as a possible solution to the challenge of avoiding hemidiaphragmatic paralysis when blocking the shoulder nerves [7]. Although the combined infraclavicular brachial plexus block-suprascapular nerve block has been successfully used for proximal humeral surgery [5], their benefits for shoulder surgery require investigation. We hypothesized that a combination of suprascapular nerve block and infraclavicular brachial plexus block would provide postoperative analgesia for patients undergoing shoulder surgery. To test this hypothesis, we performed a feasibility study in patients scheduled for shoulder surgery. The study aimed to compare infraclavicular brachial plexus block and a combination of infraclavicular brachial plexus block with suprascapular nerve block for postoperative analgesia in patients undergoing shoulder surgeries.

## Materials and methods

The present prospective randomized controlled study was conducted at King George’s Medical University, Lucknow, during the study period of January 2021 to November 2022.

Ethical considerations

The study was conducted after getting approval from the ethics committee of King George’s Medical University, Lucknow (Ref. code: VI-PGTSC-IIA/P5). Later, the study was registered in the Clinical Trials Registry of India with registration number CTRI/2022/06/043115. All the patients provided written informed consent before the start of the study.

Study criteria

A total of 62 patients of both sexes with American Society of Anaesthesiologists (ASA) physical status I/II/III, aged between 18 and 65 years, and undergoing shoulder surgery, were included in this prospective, single-blinded, randomized controlled trial. Randomization was done using the opaque sealed envelope method. Pregnant females, morbidly obese, and patients with local site infections and coagulopathy were excluded from this study. Patients were randomly divided into either of the two groups: group A patients had a brachial plexus block using the infraclavicular approach for shoulder surgeries while group B patients had a brachial plexus block using the infraclavicular approach in combination with suprascapular nerve block for shoulder surgeries.

Study procedure and assessment

A detailed pre-anesthetic assessment was done a day prior to the scheduled surgery. All patients were provided written and informed consent and were also educated about the visual analog scale (VAS) used for pain assessment. They were also explained in detail about the procedure to be done. All patients were kept nil per oral according to standard guidelines.

Patients were then taken to the operation theatre, venous cannulation was established, and standard monitors were attached. A nasal cannula was used to supply oxygen to all of the patients.

The suprascapular nerve block was performed using the anterior approach. The suprascapular nerve was identified by using an ultrasound (US) probe and a nerve stimulator (B. Braun nerve stimulator Stimuplex® HNS12, Melsungen, Germany) for stimulation. Block was given using a 5 cm 22 gauze insulated needle. When infraspinatus muscle contraction was observed at 0.5 mA current, 5 ml bupivacaine 0.375% was injected. Infraclavicular Brachial plexus block was performed using the coracoid approach. The coracoid process was identified, and the needle was introduced perpendicular to the coronal plane and parasagittally at a point located 2 cm medial and caudal to it. When muscular contraction in the hand was observed at 0.5 mA current, 30 ml bupivacaine 0.375% was injected. A nerve stimulator (Stimuplex® HNS12) was used to locate the brachial plexus along with US guidance. During injection, negative aspiration was performed after every 5 ml to avoid intravascular injection. A three-minute massage was performed to facilitate an even drug distribution. As a block adjuvant, dexamethasone (4 mg) was administered intravenously, after the completed block procedure. After injection of local anesthetic, sensory loss, and motor blockade were evaluated every 5 min. Block success was assessed after the withdrawal of the needle. The block combination was considered successful if it met the following three criteria: (a) the suprascapular nerve block had a motor score ≤4; (b) the axillary nerve sensory score was 0 or 1; (c) the musculocutaneous nerve sensory score was 0 or 1 or if the motor score was ≤4.

Subsequently, all patients underwent general anesthesia with endotracheal intubation for the procedure. Inj. fentanyl (2 ug/kg) and inj. paracetamol (PCM) 1 gm IV was given intraoperatively.

Postoperatively, all patients received 1 g paracetamol six hourly. If the visual analog scale (VAS) score for pain was >5, inj. tramadol 100 mg intravenous infusion was added as rescue analgesia. The requirement for rescue analgesia was also assessed using the VAS score. Patient satisfaction was also assessed using the numeric rating scale (NRS). NRS satisfaction is an 11-point ordinal scale assessing satisfaction with the overall management of the condition for which they sought help from the doctor, from 0 (completely dissatisfied) to 10 (completely satisfied). Higher scores depict higher satisfaction. Pain was assessed on the VAS, ranging from 0 (no pain) to 10 (worst pain).

Sample size calculation

The sample size is calculated at 90% power and based on variation in the NRS for pain for the study population using the formula [8], $n = \kappa \frac{\left (Z _{\alpha }+ Z_{\beta } \right )^{2} \left ( \sigma ^{2} \right )}{d^{2}}$, where $\sigma$ = 3.0, the IQR of the maximum score on the NRS for pain; d = 30% of median score on the NRS for pain (=6.5), the difference considered to be clinically significant; design effect $\kappa$ = 1; type I error α = 5% corresponding to a 95% confidence level, type II error β = 10% for detecting results with 90% power of study. So, the required sample size is n = 31 for each group.

Statistical analysis

Data were analyzed using the Statistical Package for the Social Sciences, Version 21 (IBM Corp., Armonk, NY, USA). Continuous variables, such as age, weight, height, and length of hospital stay, were reported as mean and standard deviation. Categorical variables were presented as frequency and percentages. The Mann-Whitney U test was applied for comparison of pain between the two groups. The chi-square test was utilized for comparing the categorical variables. A p-value of 0.05 was considered statistically significant.

## Results

**Figure 1:**
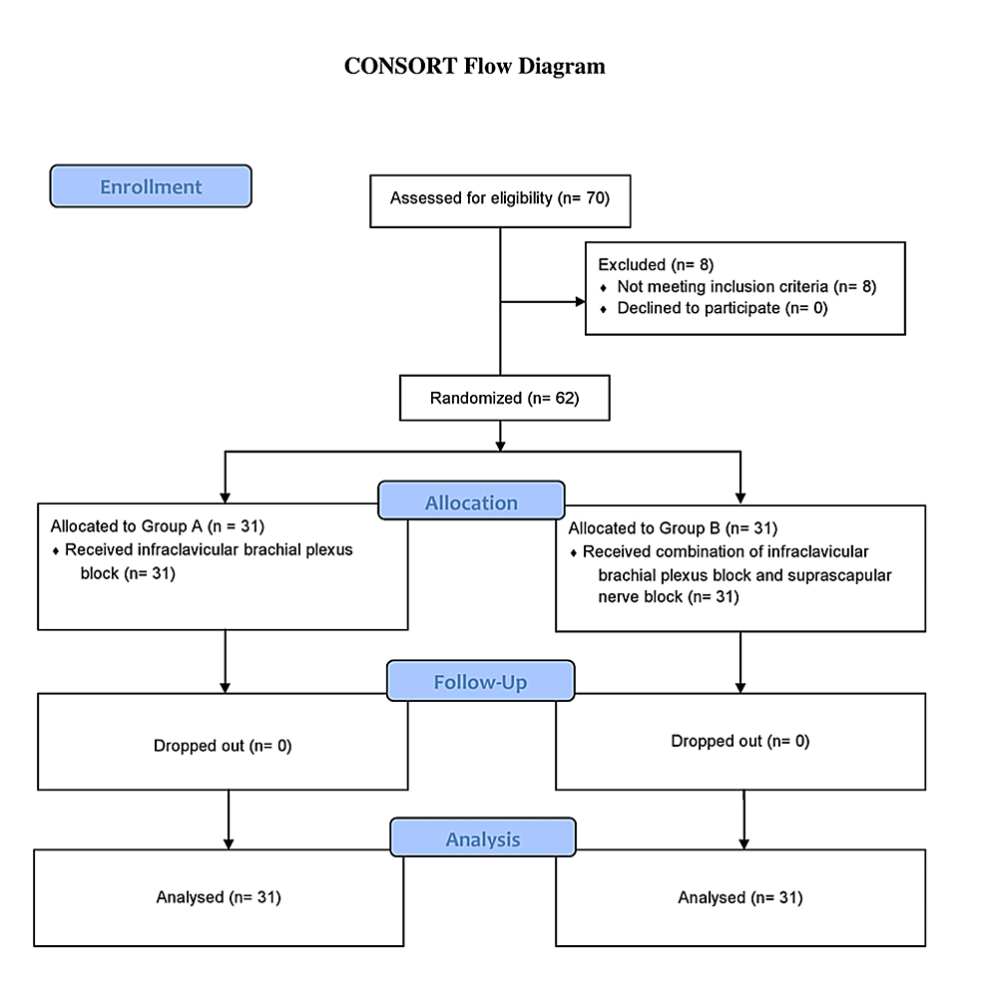
CONSORT flow diagram Group A received brachial plexus block using the infraclavicular approach for shoulder surgeries. Group B received brachial plexus block using the infraclavicular approach and suprascapular nerve block for shoulder surgeries.

Initially, 70 patients were assessed for eligibility for the study, 8 patients were excluded because of not meeting the inclusion criteria, and finally, 62 patients were analyzed. Out of 62 patients enrolled in the study, 31 (50.0%) patients were managed by brachial plexus block using the infraclavicular approach were classified as group A, and the rest (31; 50.0%) patients were managed by brachial plexus block using the infraclavicular approach in combination with a suprascapular nerve block, classified as group B (Figure 1). The groups were randomly assigned after fulfilling the inclusion criteria of the study and obtaining informed consent from the participants.

Table 1 shows that postoperative pain was significantly lower in group B as compared to group A in all periods of observation i.e. 0h (2.77±0.72 vs. 5.42±0.77), 6h (3.89±0.70 vs. 5.94±0.73), 12h (5.66±0.93 vs. 6.58±0.88), and 24h (6.16±0.80 vs. 6.74±0.90). These findings illustrate that group B who received a combination of the infraclavicular approach and suprascapular nerve block for shoulder surgeries had better pain relief than group A who received only the infraclavicular approach.

**Table 1:** Between-group comparison of pain at different time intervals p.o.: postoperative; SD: standard deviation Group A received brachial plexus block using the infraclavicular approach for shoulder surgeries. Group B received brachial plexus block using the infraclavicular approach and suprascapular nerve block for shoulder surgeries.

	Group A (n=31)	Group B (n=31)	Mann Whitney U test	
	Mean±SD	Mean±SD	‘Z’	‘p’
0h p.o.	5.42±0.77	2.77±0.72	6.846	<0.001
6h p.o.	5.94±0.73	3.89±0.70	6.764	<0.001
12h p.o.	6.58±0.88	5.66±0.93	3.641	<0.001
24h p.o.	6.74±0.90	6.16±0.80	2.962	<0.001

No complication (diaphragm palsy) was observed in all the patients except only one (3.2%) in group A; this difference was not found to be significant statistically (p=0.405). So, in terms of complications, group B who received a combined infraclavicular approach and suprascapular nerve block had fewer complications than group A who received only the infraclavicular approach for shoulder surgeries.

**Figure 2:**
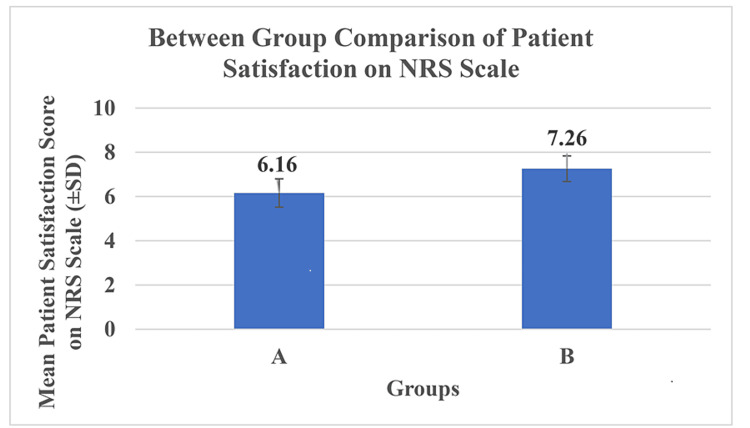
Between-group comparison of patient satisfaction on the NRS NRS: numeric rating scale Group A patients received brachial plexus block using the infraclavicular approach for shoulder surgeries. Group B received brachial plexus block using the infraclavicular approach and suprascapular nerve block for shoulder surgeries.

Figure 2 shows mean NRS score of patient satisfaction in group B (7.26±0.58) was significantly higher (p<0.001) in comparison to group A (6.16±0.64), which indicates that the infraclavicular approach combined with suprascapular nerve block in group B patients have better satisfaction than the infraclavicular approach used in group A for shoulder surgeries.

In a nutshell, the findings of the present study elucidate that group B patients who received a combination of infraclavicular approach and suprascapular nerve block show better postoperative pain relief, are more satisfied, and have fewer complications than group A patients who received only the infraclavicular approach for shoulder surgeries.

## Discussion

High levels of postoperative pain are typically experienced after shoulder surgery, necessitating narcotic analgesia for many days. Therefore, using the regional anesthesia technique is advised. For upper extremity surgery, brachial plexus block (BPB) around the clavicle has been a common technique. Currently, the gold standard for managing intraoperative and postoperative pain in patients having shoulder surgery is an interscalene brachial plexus block. However, in recent years, there has been increasing research into alternatives to the classic interscalene block due to a wide spectrum of complications, with the risk of hemi-diaphragmatic paresis of prominent interest. The innervation of the shoulder joint is provided by several nerves: subscapular, axillary, lateral pectoral, and suprascapular nerves. The infraclavicular block, as far distal as at the cord level can block the subscapular, axillary, and lateral pectoral nerves with a single injection sparing the suprascapular nerve, which must be blocked separately.

This study demonstrates postoperative analgesia assessed by pain scores (VAS score) following shoulder surgery in subjects receiving infraclavicular brachial plexus block and a combination of infraclavicular brachial plexus block with suprascapular nerve block for shoulder surgeries.

All the hemodynamic parameters (heart rate, systolic BP, diastolic BP, mean arterial pressure (MAP)) were measured before block, after block, 0h postoperatively, 1h postoperatively, 3h postoperatively, 6h postoperatively, 12h postoperatively, and 24h postoperatively. Before the block mean heart rates of group A (83.39±9.54 beats/min) and group B (83.48±10.06 beats/min) were comparable. During the rest of the periods of observation too, the heart rates of both groups were found to be comparable. The introduction of suprascapular nerve block during shoulder surgeries under brachial plexus block using the infraclavicular approach did not show any significant effect on heart rate, systolic blood pressure, diastolic blood pressure, MAP, and SpO2.

The level of pain on the VAS score was assessed immediately postoperatively (0h postoperatively), 6h postoperatively, 12h postoperatively, and 24h postoperatively. The level of pain was found to be significantly higher in group A as compared to group B at 0h postoperatively (5.42±0.77 vs. 2.77±0.72), 6h postoperatively (5.94±0.73 vs. 3.89±0.70), 12h postoperatively (6.58±0.88 vs. 5.66±0.93), and 24h (6.74±0.90 vs. 6.16±0.80). The introduction of suprascapular nerve block to the infraclavicular brachial plexus block for shoulder surgeries significantly affects the level of postoperative pain. Wiegel M et al., in their study, reported that both procedures provided good postoperative analgesia, and the mean pain level for suprascapular nerve block (SSNB) was significantly lower by 0.32 units (95% confidence interval, 0.18-0.46; P<0.001) and non-inferior, given a margin of 1.1 units; p<0.001 [9]. Within the first 24 hours, 162 (99.0%) of SSNB patients had unimpaired grip strength compared to 81 (49.0%) of interscalene BPB patients (p<0.001). The multiple primary outcomes, superior unimpaired grip strength, and non-inferior pain control were significant; p<0.001. Aliste J et al. reported that postoperatively, a blinded investigator recorded pain scores at rest at 0.5, 1, 2, 3, 6, 12, and 24 hr [10]. Compared to its infraclavicular nerve block (ICB)-suprascapular nerve block (SSB) counterpart, the interscalene brachial plexus block (ISB) group displayed lower postoperative pain scores at 30 min (difference of the medians −4; 9% confidence interval [CI] −6 to −3), required less cumulative morphine intravenous at 24 hr (difference of the means −6.1 mg;), and resulted in a higher incidence of hemi-diaphragmatic paralysis (18/20 vs 0/20 patients, respectively; p<0.001). Although postoperative pain scores at one, two, and three hours appeared lower in the ISB group, the upper bounds of the 99.0% CIs did not exceed the equivalence margin. Compared with ICB-SSB, ISB provided lower postoperative pain scores 30 min after arthroscopic shoulder surgery.

According to Pani N et al., VAS scores were evaluated at 1, 4, 6, 12, and 24h postoperatively [11]. The length of time between the first request for analgesia and the final dose needed for 24 hours after surgery, as well as patient satisfaction and any problems, were all noted. Shoulder block (SHB), which includes suprascapular block along with axillary nerve block provided equivalent analgesia to ISB in terms of postoperative VAS scores. The time to first analgesic request was 6.2±1.3h in the ISB group and 5.9±1.2h in the SHB group, which was not statistically significant. In comparison to the SHB group, complications such as subjective dyspnea and arm weakness were substantially more common in the ISB group. Additionally, the SHB group considerably outperformed the ISB group in terms of patient satisfaction levels. Due to the selective blocking of the suprascapular and axillary nerves, SHB is just as effective as ISB at relieving postoperative pain while posing fewer risks. Choi H et al. reported in their study that postoperative pain was assessed using VAS at 1, 2, 6, 12, and 24h postoperatively [12]. Supplemental analgesic use was recorded as total equianalgesic fentanyl consumption. The continuous suprascapular nerve block (C-SSNB) group showed significantly higher VAS scores at 0−1h and 1−2h after the surgery than the single shot interscalene brachial plexus block (S-ISNB) group (4.9±2.2 versus 2.3±2.2; p<0.0001 and 4.8±2.1 versus 2.4±2.3; p<0.0001, respectively). The C-SSNB group showed significantly lower VAS scores at 6−12h after the surgery than the S-ISNB group (4.1±1.8 versus.5.0±2.5; p=0.031). The analgesic effect of S-ISNB typically lasts for a maximum of six to eight hours after surgery, leading to intense rebound pain that negatively affects sleep quality and patient satisfaction [13]. As an alternative, SSNB offers a longer duration of more than six hours of postoperative pain relief [14].

In our study, at 0h postoperatively, all the patients in group B (100.0%) required PCM alone; none of them required tramadol in addition to PCM as rescue analgesia, while the vast majority of group A (93.5%) patients required PCM along with tramadol as rescue analgesia, only 6.5% required PCM alone. This difference was found significant. At 6h postoperatively too, the majority of group B (83.8%) required PCM alone as rescue analgesia, the rest required PCM plus tramadol while all the group A cases required PCR plus tramadol. This difference was found significant. At 12h postoperatively, all the patients in group A and the vast majority of group B (93.5%) required both PCM plus tramadol, and only 6.5% of group B patients required PCM alone. This difference was not significant. At 24h postoperatively, only one (3.2%) patient in group B required PCM alone, the rest required PCM plus tramadol as rescue analgesia. This difference was not significant.

Darji AV et al. defined the duration of effective postoperative analgesia as the time interval from the end of local anesthetic injection and the need for first rescue analgesia [15]. He reported that the total duration of postoperative analgesia was 459.33±20.16 minutes in group I (combined suprascapular and axillary nerve block) and 545±30.93 minutes in group S (interscalene brachial plexus block). In group I, 17.0% of patients required two doses of rescue analgesics, 30.0% of patients required three doses of required analgesics, and the rest (53.0%) of the patients required four doses of rescue analgesics within 24 hours postoperatively. Whereas in group S, 94.0% of patients required two doses of rescue analgesic, and the rest (6.0%) required three doses of rescue analgesic within 24 hours postoperatively. Hence, in comparison to interscalene brachial plexus block, combined suprascapular and axillary nerve block prolonged the duration of postoperative analgesia after shoulder surgery.

Paracetamol was prescribed as regular medication, with tramadol as rescue pain medication. However, the prescription of a wider multimodal pain medication strategy could potentially have reduced opioid consumption. Administration of adjuvants is known to prolong block duration of peripheral nerve blocks and in this study, we opted for 4 mg of dexamethasone administered intravenously. It is possible to speculate that a higher dose [16] or a combination of adjuvants [17] could have resulted in longer-lasting blocks and thus lower the total consumption of opioids. Pitambo PF et al. evaluated selective suprascapular and axillary nerve block (SG) in comparison with interscalene block (IG) [18]. After motor responses of suprascapular and axillary nerves, the SG group received 15 ml of the same substance on each nerve. General anesthesia was then administered. The mean duration of analgesia was 20.4±6.8 hours in the IG group as compared to 26.3±7.7 hours in the SG group (p<0.05). The total dose of rescue medication varied between 3 to 6 mg within 24 hours in both groups. Choi H et al. reported that the C-SSNB group required significantly higher doses of total equianalgesic fentanyl in the post-anesthesia care unit than the S-ISNB group (53.66±44.95 versus 5.93±18.25; p<0.0001) [12]. Total equianalgesic fentanyl in the ward and total equianalgesic fentanyl throughout the hospital period were similar between the groups (145.99±152.60 versus 206.13±178.79; p=0.052 and 199.72±165.50 versus 212.15±180.09; p=0.697, respectively). C-SSNB was more effective than S-ISNB at 6−12h after the surgery for postoperative analgesia after arthroscopic rotator cuff repair. Musso D et al. reported that the combination of suprascapular and infraclavicular nerve block shows an encouraging postoperative analgesic profile and a low risk for hemi-diaphragmatic paralysis after total shoulder arthroplasty [7].

In our study, diaphragmatic palsy was observed in only one (3.2%) case of group A and none of group B. No other complication was observed in any of the patients during the study period. Similarly, Musso et al. conducted a study that showed that one patient (5.0%) was diagnosed with hemidiaphragmatic paralysis, which was confirmed by a chest X-ray [7]. Hemidiaphragmatic function resumed when the local anesthetic effect had worn off.

Patient satisfaction is an increasingly important dimension of health outcome research. A simple NRS measure of satisfaction is sufficient to capture satisfaction with current management of the condition, and more feasible and accepted for use both by the doctor and the patients. In our study, patient satisfaction was evaluated on the NRS on a scale of 0-10, range of patient satisfaction on the NRS scale in the study population ranged from 5-8, the median patient score of group A was 6.0 while that of group B was 7.0. A significantly higher patient satisfaction score was observed in group B (7.26±0.58) than in group A (6.16±0.64). This might be due to better pain control in group B than in group A, which led to higher levels of satisfaction in group B patients.

Shoulder surgeries are preferably being done under regional anesthesia nowadays. There are various approaches to block the brachial plexus to provide analgesia during and after procedures. However, due to complications like diaphragmatic palsy and Horner’s syndrome with the interscalene and supraclavicular blocks, alternative approaches must be looked for. Hence, in our study, we found that the combination of infra-clavicular block and suprascapular block was better in terms of providing a longer duration of post-operative analgesia and patient satisfaction and reduced the requirement for rescue analgesia in the postoperative period.

Limitations of the study

The study had a relatively smaller sample size. A larger sample size and multicentric studies should be done for better validation of results.

## Conclusions

In conclusion, this novel combination of peripheral nerve blocks provides surgical analgesia and satisfactory postoperative analgesia for patients scheduled for shoulder surgery. Combined suprascapular and infraclavicular nerve block provided a prolonged duration of postoperative analgesia as compared to infraclavicular brachial plexus block alone following shoulder surgery. The combination of infraclavicular and suprascapular nerve blocks displays a positive postoperative analgesic profile with less use of rescue analgesic doses and better patient satisfaction after shoulder surgery. However, randomized controlled trials with larger sample sizes and multicentric studies should be done in the future to validate these results.
